# Scaling Up Testing for Human Immunodeficiency Virus Infection Among Contacts of Index Patients — 20 Countries, 2016–2018

**DOI:** 10.15585/mmwr.mm6821a2

**Published:** 2019-05-31

**Authors:** Arielle Lasry, Amy Medley, Stephanie Behel, Mohammed I. Mujawar, Meagan Cain, Shane T. Diekman, Jacqueline Rurangirwa, Eduardo Valverde, Robert Nelson, Simon Agolory, Achamyeleh Alebachew, Andrew F. Auld, Shirish Balachandra, Sudhir Bunga, Thato Chidarikire, Vinh Q. Dao, Jacob Dee, L.E. Nicole Doumatey, Edington Dzinotyiweyi, Eric J. Dziuban, K. Alexandre Ekra, William B. Fuller, Amy Herman-Roloff, Nely B. Honwana, Nompumelelo Khanyile, Evelyn J. Kim, S. Francois Kitenge, Romel S. Lacson, Peter Loeto, Samuel S. Malamba, André H. Mbayiha, Alemayehu Mekonnen, Mirtie G. Meselu, Leigh Ann Miller, Goabaone P. Mogomotsi, Mary K. Mugambi, Lloyd Mulenga, Jane W. Mwangi, Jonathan Mwangi, Amassanh A. Nicoué, Mtemwa K. Nyangulu, Ismelda C. Pietersen, Puleng Ramphalla, Chanie Temesgen, Alfredo E. Vergara, Stanley Wei

**Affiliations:** ^1^Division of Global HIV and Tuberculosis, Center for Global Health, CDC; ^2^Federal Ministry of Health, Ethiopia; ^3^National Department of Health, South Africa; ^4^Ministry of Health and Social Services, Namibia; ^5^Ministry of Health and Wellness, Botswana; ^6^National AIDS and STIs Control Programme, Kenya; ^7^Ministry of Health, Zambia.

In 2017, the Joint United Nations Programme on HIV/AIDS (UNAIDS) estimated that worldwide, 36.9 million persons were living with human immunodeficiency virus (HIV) infection, the virus infection that causes acquired immunodeficiency syndrome (AIDS). Among persons with HIV infection, approximately 75% were aware of their HIV status, leaving 9.4 million persons with undiagnosed infection (*1*). Index testing, also known as partner notification or contact tracing, is an effective case-finding strategy that targets the exposed contacts of HIV-positive persons for HIV testing services. This report summarizes data from HIV tests using index testing in 20 countries supported by CDC through the U.S. President’s Emergency Plan for AIDS Relief (PEPFAR) during October 1, 2016–March 31, 2018. During this 18-month period, 1,700,998 HIV tests with 99,201 (5.8%) positive results were reported using index testing. The positivity rate for index testing was 9.8% among persons aged ≥15 years and 1.5% among persons aged <15 years. During the reporting period, HIV positivity increased 64% among persons aged ≥15 years (from 7.6% to 12.5%) and 67% among persons aged <15 years (from 1.2% to 2.0%). Expanding index testing services could help increase the number of persons with HIV infection who know their status, are initiated onto antiretroviral treatment, and consequently reduce the number of persons who can transmit the virus.

To end the HIV epidemic by 2020, UNAIDS set multiple targets, including increasing to 90% the percentage of persons with HIV infection who knew their HIV status (*2*). Results from population-based HIV impact assessments in 10 African countries indicated that, as of 2018, the percentage of persons with HIV infection who knew their status ranged from 37% in Côte d’Ivoire to 86% in Namibia (*3*).

Since 2003, PEPFAR has provided approximately $72 billion to implement HIV testing and treatment programs in 37 countries and regions with high HIV prevalence (*4*). PEPFAR funds are administered through multiple U.S. governmental agencies, including CDC, that support international and local organizations and governments for HIV-related program implementation. In 2017, PEPFAR supported 85.5 million HIV rapid tests and linked 14 million adults and children to antiretroviral treatment (*5*).

Because HIV testing service resources from donors and governments are scarce, targeted strategies are needed to reach persons with undiagnosed HIV infection. In index testing, also known as partner notification or contact tracing, HIV-positive index patients voluntarily identify their sexual and needle-sharing partners and biologic children. Partners and children of index patients, who might have been exposed to HIV, are then contacted by the index patient or the provider, and those whose HIV infection status is not known are offered HIV testing services. Studies have demonstrated the effectiveness and cost-effectiveness of index testing as a strategy for identifying HIV-positive adults and children (*6*–*9*).

HIV program implementing partners supported by PEPFAR collect and report data for performance monitoring and evaluation purposes on a quarterly basis in accordance with the U.S. fiscal year (October 1–September 30). The primary HIV testing indicator is the number of persons who have received HIV testing services, categorized by HIV result, age group, sex, and testing service delivery approach. Age group categories are classified as <1 year, 1–9 years, 10–14 years, 15–19 years, 20–24 years, 25–49 years, and ≥50 years. Sex is not reported for children aged <10 years. Delivery approaches for HIV testing services include 1) community-based testing in mobile clinics; 2) voluntary drop-in centers; 3) facility-based provider-initiated testing in tuberculosis, sexually transmitted infection, outpatient, and antenatal clinics; 4) testing in hospital emergency and inpatient departments; and 5) since October 1, 2016, index testing.

This report includes the six most recent fiscal quarters for which index testing data were available (October 1, 2016–March 31, 2018). Among 33 countries reporting index testing data during this period, seven countries (Angola, China, Dominican Republic, El Salvador, Guyana, Honduras, and Thailand) that reported <1,000 persons tested using index testing and four countries (India, Kazakhstan, Kyrgyzstan, and Tajikistan) that reported <500 tests during October 1, 2017–March 31, 2018 were excluded from this analysis. Also excluded were Nigeria and Ukraine in response to requests from the country offices. The number of HIV tests reported using index testing and the percentage of positive tests among different demographic groups in CDC-supported PEPFAR programs in 20 countries* were summarized for this report.

From October 1, 2016, to March 31, 2018, CDC-supported implementing partners reported a total of 1,700,998 persons tested for HIV using index testing among the 20 countries evaluated, including 889,599 (52%) persons aged ≥15 years and 799,976 persons aged <15 years (Table 1). Overall, 99,201 (5.8%) persons were reported as HIV-positive, including 87,266 persons aged ≥15 years and 11,814 persons aged <15 years. Index testing from three countries (Kenya, Mozambique, and Tanzania) accounted for more than half of all HIV tests and positive results reported. By age group, 9.8% of HIV test results among persons aged ≥15 years and 1.5% among persons aged <15 years were positive. The rate of HIV positivity by country ranged from 0.7% to 24.5% among persons aged <15 years and from 2.8% to 29.1% among persons aged ≥15 years.

**TABLE 1:** Number of human immunodeficiency virus (HIV) index tests performed,* and number and percentage of HIV-positive results, by age group and country — 20 countries, October 1, 2016–March 31, 2018^†^

Country	Persons aged ≥15 yrs		Persons aged <15 yrs		Total^§^	
	No. of HIV tests	No. of HIV-positive results (%)	No. of HIV tests	No. of HIV-positive results (%)	No. of HIV tests	No. of HIV-positive results (%)
Mozambique	190,474	34,876 (18.3)	283,755	4,395 (1.5)	**474,801**	**39,351 (8.3)**
Kenya	174,839	7,616 (4.4)	212,676	1,435 (0.7)	**387,599**	**9,053 (2.3)**
Tanzania	101,004	6,784 (6.7)	59,206	1,012 (1.7)	**160,920**	**7,816 (4.9)**
Zambia	95,896	5,949 (6.2)	56,575	862 (1.5)	**152,474**	**6,814 (4.5)**
Namibia	52,580	5,234 (10.0)	211	4 (1.9)	**52,791**	**5,238 (9.9)**
Côte d'Ivoire	36,178	4,130 (11.4)	46,599	997 (2.1)	**82,785**	**5,127 (6.2)**
South Africa	40,294	4,985 (12.4)	1,991	138 (6.9)	**42,285**	**5,123 (12.1)**
Ethiopia	68,999	3,309 (4.8)	48,670	839 (1.7)	**117,731**	**4,154 (3.5)**
Cameroon	27,104	3,437 (12.7)	18,182	293 (1.6)	**45,371**	**3,735 (8.2)**
Zimbabwe	10,897	2,374 (21.8)	14,071	640 (4.5)	**24,968**	**3,014 (12.1)**
Eswatini^¶^	6,917	1,881 (27.2)	169	11 (6.5)	**7,088**	**1,892 (26.7)**
Malawi	8,561	1,426 (16.7)	2,999	144 (4.8)	**11,602**	**1,573 (13.6)**
DRC	7,949	1,078 (13.6)	6,723	364 (5.4)	**14,672**	**1,442 (9.8)**
Uganda	26,990	1,140 (4.2)	16,277	160 (1.0)	**43,267**	**1,300 (3.0)**
Lesotho	9,626	988 (10.3)	18,421	145 (0.8)	**28,056**	**1,133 (4.0)**
Haiti	7,997	616 (7.7)	761	34 (4.5)	**18,563**	**652 (3.5)**
South Sudan	1,064	310 (29.1)	895	219 (24.5)	**1,959**	**529 (27.0)**
Rwanda	13,852	381 (2.8)	11,535	114 (1.0)	**25,424**	**495 (1.9)**
Vietnam	5,725	395 (6.9)	26	1 (3.8)	**5,752**	**396 (6.9)**
Botswana	2,653	357 (13.5)	234	7 (3.0)	**2,890**	**364 (12.6)**
**Total**	**889,599**	**87,266 (9.8)**	**799,976**	**11,814 (1.5)**	**1,700,998**	**99,201 (5.8)**

**Abbreviation:** DRC = Democratic Republic of the Congo.

* Testing was supported by CDC through the U.S. President’s Emergency Plan for AIDS Relief.

^†^ Among 33 reporting countries, seven reporting <1,000 tests (Angola, China, Dominican Republic, El Salvador, Guyana, Honduras, and Thailand) and four reporting <500 tests during October 1, 2017–March 31, 2018 (India, Kazakhstan, Kyrgyzstan, and Tajikistan) were excluded. Also excluded were Nigeria and Ukraine, in response to country office requests.

^§^ Total might differ from sum of persons aged ≥15 years and persons aged <15 years because of incompleteness of reporting by age group.

^¶^ The reporting definition for index testing used by Eswatini has changed and might affect the results.

During the six fiscal quarters covered by this report, the number of persons tested for HIV using index testing among the 20 countries increased from 166,108 to 356,573 from the first to sixth quarter (Figure). During this period, the number of positive results more than tripled from 8,186 to 27,893. The quarterly rates of HIV positivity increased from 4.9% to 7.8% overall, from 7.6% to 12.5% among persons aged ≥15 years, and from 1.2% to 2.0% among persons aged <15 years (Figure).

**FIGURE:**
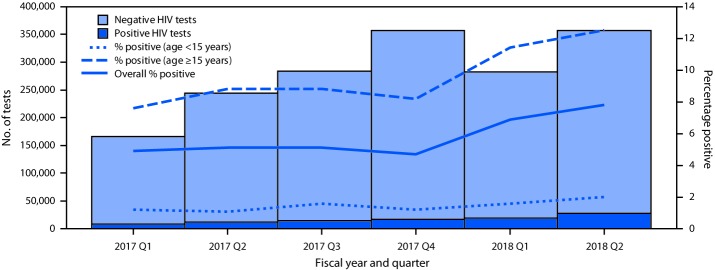
Percentage of quarterly human immunodeficiency virus (HIV) index tests with HIV-positive results, overall and by age group — 20 countries,* October 1, 2016–March 31, 2018 * Botswana, Cameroon, Côte d’Ivoire, Democratic Republic of the Congo, Eswatini, Ethiopia, Haiti, Kenya, Lesotho, Malawi, Mozambique, Namibia, Rwanda, South Africa, South Sudan, Tanzania, Uganda, Vietnam, Zambia, and Zimbabwe.

Among the 865,126 persons aged ≥15 years tested for whom sex was reported, 55% were females and 45% were males (Table 2). HIV index testing positivity rates were lowest among males and females aged 10–14 years (1.1% and 1.3%, respectively) and highest among men and women aged 25–49 years (13.0% and 13.5%, respectively). Overall, the mean rate of index testing HIV positivity was 7.5% among females and 6.8% among males.

**TABLE 2:** Number of human immunodeficiency virus (HIV) index tests performed* and number and percentage of HIV-positive results, by age group and sex — 20 countries,^†^ October 1, 2016–March 31, 2018

Characteristic	No. of HIV tests	No. of HIV-positive tests (%)
**Age group (yrs)**		
<1	19,281	597 (3.1)
1–9	466,534	7,333 (1.6)
10–14	294,194	3,625 (1.2)
15–19	207,958	6,582 (3.2)
20–24	158,036	10,563 (6.7)
25–49	434,553	57,559 (13.2)
≥50	64,579	6,279 (9.7)
Unknown	55,863	6,663 (11.9)
**Sex**		
Female	628,669	47,109 (7.5)
Male	497,309	33,982 (6.8)
Unknown	485,815	7,930 (1.6)
**Sex/Age group (yrs)**		
**Female**		
10–14	156,571	2,066 (1.3)
15–19	116,806	4,281 (3.7)
20–24	97,267	7,402 (7.6)
25–49	226,788	30,598 (13.5)
≥50	31,237	2,762 (8.8)
All females aged ≥15 years	472,098	45,043 (9.5)
**Male**		
10–14	137,623	1,559 (1.1)
15–19	91,152	2,301 (2.5)
20–24	60,769	3,161 (5.2)
25–49	207,765	26,961 (13.0)
≥50	33,342	3,517 (10.5)
All males aged ≥15 years	393,028	35,940 (9.1)
**Total**	**1,700,998**	**99,201 (5.8)**

* Testing was supported by CDC through the U.S. President’s Emergency Plan for AIDS Relief.

^†^ Botswana, Cameroon, Côte d’Ivoire, Democratic Republic of the Congo, Eswatini, Ethiopia, Haiti, Kenya, Lesotho, Malawi, Mozambique, Namibia, Rwanda, South Africa, South Sudan, Tanzania, Uganda, Vietnam, Zambia, and Zimbabwe.

## Discussion

From October 1, 2016, to March 31, 2018, CDC-supported PEPFAR partners in 20 countries reported administering a total of 66 million HIV tests over all approaches, 70% of which occurred in facility settings and 30% in community-based settings. The HIV positivity rate using all testing approaches was 1.0% among persons aged <15 years and 4.1% among persons aged ≥15 years (*10*). The rates of positivity reported through index testing were higher, 1.5% among persons aged <15 years and 9.8% among persons aged ≥15 years. The variation in HIV testing positivity among countries is likely related to differences in coverage with HIV services; in countries where coverage is low, the likelihood of eliciting HIV-positive contacts who did not know their status is higher than in countries where coverage with HIV services is high. Data indicate that both the volume and efficiency of the index testing approach are increasing, and index testing represented 2.4% of all tests reported among HIV testing delivery services (*10*). This suggests that index testing is a promising strategy for identifying HIV-positive persons, particularly in countries with low coverage with HIV services.

The percentage of index testing HIV positivity was similar among men and women; however, percentages for adults and children varied widely. Children are likely to have acquired HIV infection perinatally, and the rate of index testing positivity among persons aged <15 years is much lower than that of persons in older age groups, who likely acquired HIV from sexual and needle-sharing partners.

The findings in this report are subject to at least three limitations. First, the data might include HIV-positive persons aware of their status who chose to retest or HIV-negative persons who tested more than once during the reporting period. Removing these duplicate testing events is not possible because routine data are reported in aggregate and many of the countries do not have unique population identifiers to support the deduplication of testing records. Second, index testing has been recently prioritized, as evidenced by the December 2016 World Health Organization guidelines on HIV self-testing and partner notification,^†^ and countries are at varying stages in index testing implementation, which might explain the wide range in HIV positivity rates. Also, reporting of index testing as a service delivery approach was introduced by PEPFAR in October 2016, and implementing partners might need an adjustment period (e.g., to avoid misclassifying nonexposed contacts such as neighbors and other household members). Thus, the data might overreport or underreport the numbers tested and positivity rate through the index testing modality in some countries. Finally, persons aged 25–49 years were categorized in one group, and differences in the rates of positivity within that group cannot be evaluated. Because the highest rates of HIV positivity were in the 25–49 year age group, evaluation of narrower age groups might provide further insights. As of October 2018, PEPFAR-funded countries have been advised to report 5-year age increments within the 25–49 years age group.

HIV testing among contacts of known index patients is an effective approach to identifying HIV-positive persons, particularly among persons aged 25–49 years. In addition, men accounted for 45% of adults tested through index testing, which suggests that index testing is an efficient method for identifying HIV-positive men, who might be missed with other approaches. Scaling up index testing as part of the overall HIV testing services strategy could help increase the number of HIV-positive persons who know their status, are initiated onto antiretroviral treatment, and consequently reduce the number of persons who can transmit the virus.

**Summary**

**What is already known about this topic?**

Index testing identifies the exposed partners and biologic children of persons with diagnosed human immunodeficiency virus (HIV) infection and offers these contacts HIV testing services.

**What is added by this report?**

From October 2016 to March 2018, both the number of persons tested for HIV and the number who received a diagnosis of HIV infection using index testing increased in 20 CDC-supported countries. With an HIV positive rate that is more than twice that of all HIV testing approaches combined, index testing was found to be a more efficient approach to HIV case finding.

**What are the implications for public health practice?**

Expanding index testing services could help increase the number of HIV-positive persons who know their HIV infection status, increase the number who receive antiretroviral treatment, and, as a result, reduce the number of persons who can transmit the virus.

## Footnotes

* Botswana, Cameroon, Côte d’Ivoire, Democratic Republic of the Congo, Eswatini, Ethiopia, Haiti, Kenya, Lesotho, Malawi, Mozambique, Namibia, Rwanda, South Africa, South Sudan, Tanzania, Uganda, Vietnam, Zambia, and Zimbabwe.

† https://www.who.int/hiv/pub/vct/hiv-self-testing-guidelines/en/.
